# Glucose control and variability assessed by continuous glucose monitoring in patients with type 1 diabetes and diabetic kidney disease

**DOI:** 10.3892/br.2024.1901

**Published:** 2024-12-02

**Authors:** Aleksejs Fedulovs, Jana Janevica, Lelde Kruzmane, Jelizaveta Sokolovska

**Affiliations:** 1 Faculty of Medicine and Life Sciences, University of Latvia, Riga LV-1004, Latvia; 2 Outpatient Department, Pauls Stradins Clinical University Hospital, Riga LV-1002, Latvia

**Keywords:** type 1 diabetes, continuous glucose monitoring, glucose variability, diabetic kidney disease

## Abstract

Continuous glucose monitoring (CGM) has emerged as a superior method to glycated hemoglobin (HbA1c) monitoring for glycemic control assessment in type 1 diabetes (T1D). The association between CGM parameters and diabetic kidney disease (DKD) has not been extensively researched. The aim of the present study was to compare CGM metrics between patients with stable and progressive DKD and T1D. A cross-sectional study was performed with 75 patients with T1D, of which 28 had progressive DKD, defined as an estimated glomerular filtration rate decrease of ≥3 ml/min/year or an increased albuminuria stage over the median follow-up time of 7.46 (6.50-8.16) years. FreeStyle Libre ProiQ Sensors were used for CGM. Insulin sensitivity was calculated according to the estimated glucose disposal rate (eGDR) formula. The results revealed that as compared with subjects with stable DKD, individuals with progressive DKD exhibited a higher average glucose level (P=0.03), spent more time above the target range (P=0.05), less time in time in range (TIR; P=0.03), had a higher median estimated HbA1c (P=0.02) and glucose management indicator (P=0.03), as well as a longer duration of hypoglycemic events (P=0.03). There were no differences in compliance levels and recognition of hypoglycemia between the DKD study groups. Differences in correlation patterns between CGM parameters in patients with stable and progressive DKD were observed. For example, glucose variability was significantly positively correlated with TIR in subjects with DKD (Ρ=0.390; P=0.04) but not in individuals without DKD. The progression of DKD was statistically significantly associated with several CGM parameters in multivariate logistic regression models. Collectively, associations between CGM metrics and DKD status were demonstrated in patients with T1D. The findings of the present study indicate the necessity for regular CGM in patients with progressive DKD for improvement of their glycemic control and DKD outcomes but also call for the development of a personalized approach to CGM data interpretation and establishing therapeutic targets in these subjects.

## Introduction

Type 1 diabetes (T1D) is a chronic autoimmune condition characterized by absolute insulin deficiency and hyperglycemia. Chronic hyperglycemia poses the risk of serious vascular complications, including diabetic kidney disease (DKD). DKD is characterized by a gradual decline in kidney function and an increased risk of advancing to end-stage renal disease (ESRD) (1).

Glycated hemoglobin (HbA1c), a traditional marker of long-term glycemic control, has been demonstrated to be one of the main risk factors for DKD progression (1,2). However, with the increasing use of continuous glucose monitoring (CGM) in T1D management, other metrics of glucose control such as time in range (TIR), coefficient of variation (CV), time above range (TAR), time below range (TBR), glucose management indicator (GMI), average glucose, estimated A1C, hypoglycemia events and duration were identified to better characterize diabetes control. Previous studies indicate that glucose variability (GV) characterized by CV in CGM, or fluctuations of blood glucose levels, play a significant role in long-term T1D outcomes (3,4). The main mechanism, which is implicated in the increased risk of complications associated with GV, is considered to be oxidative stress (5-8).

Some research groups have identified associations between HbA1c and fasting GV and DKD (9). However, HbA1c variability assessment does not provide insight into daily and hourly glucose fluctuations (10), which can be evaluated using a CGM approach. Regrettably, there has been limited research on the associations between DKD progression and CGM metrics or multiple daily glucose measurements (11) with conflicting results. For example, there is data from the Diabetes Control and Complications Trial (DCTT) study reporting GV computed from 7-point daily glucose measurement profiles that were not linked to an increased risk of diabetic nephropathy in patients with T1D (12). Conversely, in advanced DKD in the dialysis setting, increased GV assessed by CGM was observed in patients with T1D and T2D (13). Notably, ESRD represents the final stage of DKD. In addition, dialysis may be associated with variations in glycemic control during dialysis and non-dialysis days (14,15). Another study reported associations between lower TIR and composite microvascular complications (MVC; presence of neuropathy, retinopathy, or nephropathy) in 515 subjects with T1D (16). However, studies reporting CGM parameters in patients with different DKD stages and linking CGM parameters with DKD progression in T1D are lacking.

There are multiple reasons for increased GV in T1D. Postprandial glucose levels are determined by the rate of nutrient delivery into the intestine, absorption of nutrients from the small intestine, and the metabolism of the absorbed nutrients by the liver. Thus, GV might be influenced by the rate of gastric emptying, chronic gastro-intestinal diseases, autonomic neuropathy and errors in adjustment of insulin treatment (17). In addition, impaired kidney function is a risk factor for hypoglycemia due to a possible impairment in kidney gluconeogenesis (18) which might impact GV and CGM results in general.

To summarize, it is currently uncertain whether individuals with progressive DKD, including its early stages, exhibit distinct CGM outcomes compared with those with T1D but without DKD. It is argued that such information is crucial for several reasons: Firstly, to investigate the relationship between DKD progression and real-time glucose management; secondly, to establish that patients with advancing diabetes-related complications should be prioritized for CGM to enhance glucose regulation and mitigate the advancement of complications; and thirdly, to identify CGM profile characteristics of DKD for better patient surveillance and identification of risk factors associated with increased GV.

Recognizing this gap in research, the present study aimed to explore the associations between CGM parameters and DKD progression in patients T1D, for enhanced patient care and management strategies for the future.

## Materials and methods

### Patient recruitment and ethical approval

The present study is part of the longitudinal LatDiane study, which commenced in 2013, and is a participant in the international InterDiane consortium. LatDiane focuses on recruiting adult patients with T1D who were diagnosed before the age of 40, started insulin treatment within one year of diagnosis, and have C-peptide levels <0.3 nmol/l. Patients with chronic kidney disease unrelated to DKD are excluded. The evaluations are performed based on the medical records of patients (19). Follow-up visits occur every three years. The study protocol for the overall LatDiane study and the sub-study reported herein have received approvals from the Latvian Central Ethics Committee (Riga, Latvia) under permission nos. 01-29.1/3 (dated, 10.07.2013), Nr.A-17/19-10-17 (dated, 17.10.2019), and Nr. 01-29.1.2/927 (dated, 09.12.2020). Biobanking, and sample storage were conducted following the procedures of the Genome Database of the Latvian population and are detailed in previous studies (20,21). The present study adheres to the principles outlined in the 1964 Declaration of Helsinki and its subsequent amendments. Prior to inclusion in the study, written informed consent was obtained from all participants. Recruitment for this study took place between October 1, 2021 and July 30, 2022, at the University of Latvia (Riga, Latvia). Information regarding the study was disseminated through the University of Latvia's website and social media channels. The inclusion criteria for this study required a minimum T1D duration of 8 years and available data on the progression of DKD, including at least three measurements of serum creatinine and three measurements of albuminuria between the baseline visit of the LatDiane study (2013-2021) and the present study (Fig. S1). The rationale for selecting participants with a minimum duration of diabetes of at least 8 years is outlined as follows: In T1D, the screening (yearly albuminuria and serum creatinine measurements) for MVC including DKD commences 5 years after the onset of diabetes (22), and in order to calculate the eGFR slope and assess the eGFR decline, one of the metrics to define whether DKD is progressing, at least 3 additional years are required, thus totaling 8 years. The definitions for progressive DKD were used as described in a study by Colombo *et al* (23).

The exclusion criteria included pregnancy, a history of inflammatory bowel disease (such as Crohn's disease or ulcerative colitis) and celiac disease (detected through serum transglutaminase IgA screening), as well as a recent acute intestinal infection within 2 months, clinical signs of acute inflammation, and fever. On the study day, patients underwent investigations for the collection of anthropometric measures, completion of questionnaires, CGM sensor installation and blood sample collection.

### Clinical investigation and monitoring of diabetic complications and co-morbidities

Blood pressure readings were obtained for all patients. Those with a systolic blood pressure of 140 mmHg (18.7 kPa) or higher, a diastolic blood pressure of 90 mmHg (12.0 kPa) or higher, or a history of antihypertensive drug use were classified as having arterial hypertension. Smoking status was determined through self-reported data via a questionnaire, with the ‘smokers’ category including patients currently smoking at least one cigarette daily.

The assessment of cardiovascular disease (CVD) and diabetes-related complications, such as retinopathy, neuropathy, and DKD, was conducted through a review of medical records. CVD was defined based on a history of acute myocardial infarction, coronary bypass or percutaneous transluminal coronary angioplasty, stroke, amputation, or peripheral vascular disease. The determination of albuminuria involved calculating the albumin-to-creatinine ratio from two out of three collected morning spot urine samples. The estimated glomerular filtration rate (eGFR) was calculated according to the Chronic Kidney Disease Epidemiology Collaboration (CKD-EPI) equation (24)

In this equation, the creatinine value is measured in milligrams per deciliter (mg/dl), and the age is expressed in years.

ESRD was defined as an eGFR <15 ml/min/1.73 m², requiring dialysis or kidney transplantation. Progressive DKD was defined as an eGFR decline exceeding 3 ml/min/1.73 m² per year utilizing at least three serum creatinine measurements, or an increase in the stage of albuminuria during the follow-up period (23).

Insulin resistance was estimated via the estimated glucose disposal rate (eGDR) formula. The lower the eGDR, the higher the insulin resistance.

eGDR=24.4-12.97 x Waist/Hips-3.39 x Hypertension-0.6 x HbA1c%

If the blood pressure was ≥140/90 mmHg and/or the patient regularly took antihypertensive medication, hypertension was indicated as 1; otherwise, it was indicated as 0(25).

### Blood samples

Blood samples were collected through venipuncture and, along with morning spot urine samples, were promptly sent to a certified clinical laboratory for measurement of clinical analytes.

### Monitoring of compliance level to diabetes self-management

Diabetes diaries were used to monitor compliance levels. Patients were asked to document at least four capillary blood glucose levels each day, carbohydrates consumed, insulin doses administered, and other glucose-influencing factors for 14 days. Patients measured glucose levels at waking, before meals, 2 h post-meal, and before bed, with an additional 2:00 a.m. check every third day. The compliance level was assessed based on the number of entries made for the necessary parameters over a 14-day period. The compliance levels were categorized as follows:

• Low: <6 days with necessary entries

• Moderate: 6-10 days with necessary entries

• High: ≥10 days with necessary entries

### CGM

FreeStyle Libre ProiQ Sensors (diagnostic or ‘blinded’ glucose sensors; Abbott GmbH) were used for CGM. Participants had to wear the sensor for 14 days. Average glucose, CV, estimated HbA1c, glucose management indicator (GMI), percentage of TAR (%TAR), percentage of TIR (%TIR), percentage of TBR (%TBR), and low glucose events were analyzed. TIR was defined as the percentage (%) of time when glucose levels were between 3.9 and 10 mmol/l, according to the recommendations applicable to most subjects with T1D (10).

Out of the 78 subjects initially recruited, 75 had glucose sensor data after 14 days of CGM. Therefore, data on 75 subjects were analyzed.

### Statistical analysis

Statistical analyses were performed using SPSS version 27 (IBM Corp.), and statistical visualizations were created using the open-source software R version 4.3.1(26), along with the ggplot2 package version 3.5.1(27).

The one-sample Kolmogorov-Smirnov test was employed along with histogram analysis to assess the normality of the variables of the study. All continuous variables were found to deviate from a normal distribution. Descriptive statistical analyses were performed on all variables in the study. Categorical variables were presented as counts and percentages of the total samples of the study. Continuous variables were summarized using median values and interquartile ranges (IQR). Univariate analyses were conducted to compare socio-demographic variables between the study groups. Categorical variables were analyzed using the Chi-square test, and continuous variables were evaluated using the Mann-Whitney U test.

Spearman's correlation analysis was conducted to examine the relationships between CGM parameters, kidney and liver function markers, body mass index (BMI), T1D disease duration, and eGDR within each study group, with results presented as Spearman's correlation coefficients (95% CI).

Multiple binary logistic regression models were constructed to assess the association between DKD progression and CGM parameters. Models were adjusted for clinically relevant variables including sex, BMI, age, and diabetes duration and tested for fit by performing the Hosmer-Lemeshow test. P<0.05 was considered to indicate a statistically significant difference.

## Results

### Description of the whole study cohort

The present study included a sample of 75 patients with T1D from the longitudinal LatDiane cohort. The cohort was followed for a median duration of 7.46 years (6.50-8.16 years). The majority of included subjects were women (n=44, 58.67%). The median age of the participants was 44.00 years (34.00-53.00 years), and their BMI was 24.50 kg/m^2^ (22.95-28.30 kg/m^2^). Most of the participants were non-smokers 41 (54.67 %), and 44 (58.67%) had hypertension. The median duration of diabetes in the group was 25.00 years (16.50-33.00 years), and the HbA1c level was 8.20% (7.30-9.55%). Among the subjects, 39 (52.00%) had retinopathy, 22 (29.33%) had autoimmune thyroid disease, 14 (18.67%) had other autoimmune diseases, and 14 (18.67%) had CVD. Out of the total subjects included in the study, 10 individuals (13.33%) were found to have macroalbuminuria. Data are summarized in Table SI. At the time of the study, 28 subjects had progressive DKD.

### Description of the study groups

The study groups, categorized based on the presence of stable DKD or progressive DKD, did not differ in median age, sex distribution, median BMI and median HbA1c. Subjects with progressive DKD had a higher prevalence of hypertension (P=0.02) and a longer history of diabetes (P=0.01) compared with patients with stable DKD. Additionally, significant differences were observed in the presence of retinopathy, CVD, and albuminuria between the two groups (all P<0.01). Triglyceride levels were higher in the progressive DKD group compared with the stable DKD group (P=0.04). As anticipated, eGFR was lower in the progressive DKD group compared with the stable DKD group (P=0.02). The dialysis occurrence 5 (17.9%) and kidney transplantation occurrence 7 (25.0%), both markers of ESRD, were more frequent in the progressive DKD group. Characteristics of the groups are summarized in Table I.

### CGM metrics and DKD

In total, 75 individuals had available CGM data after sensor wear for 14 days. The median duration of CGM sensor wear among T1D patients was 15 days (15.00-15.00 days). The cohort exhibited a median average glucose level of 9.50 mmol/l (8.10-11.40 mmol/l), with a CV of 39.59% (35.67-43.67%). The GMI was 7.40% (6.80-8.20%). The distribution of glucose levels revealed that the median TAR was 43.00% (27.00-59.00%), TIR was 51.00% (37.00-66.00%), and the TBR was 4.00% (2.00-10.00%). Low glucose events were quantified at 9.00 (4.00-15.00), with the median duration of 110 min (76.00-143.50 min). The estimated A1C for the cohort was 7.60% (6.65-8.75%). Overnight hypoglycemia was prevalent in 55 participants during the monitoring period, constituting 74.32% of the cohort. Data are summarized in Table SII.

The analysis of CGM data between the study groups revealed that individuals with progressive DKD had higher average glucose levels, while those with stable DKD exhibited lower results (P=0.03). TAR was increased in individuals with progressive DKD compared with stable DKD (P=0.05). Conversely, the stable DKD group had a higher percentage of TIR compared with individuals with progressive DKD (P=0.03). Individuals with progressive DKD had longer median duration of low glucose events compared with those with stable DKD (P=0.03). Furthermore, estimated A1C levels were higher in the progressive DKD group compared with the stable DKD group (P=0.02). Similarly, GMI was higher in the progressive DKD group compared with the stable DKD group (P=0.03). The observed differences across a range of factors are summarized in Table II.

### Compliance to diabetes self-management and DKD

In total, 74 participants provided diabetes diary data. To elucidate the distribution of compliance levels (low, moderate, high) among the cohort a comprehensive analysis was conducted. The findings revealed that 15 individuals (20.27%) exhibited low levels of compliance, 14 individuals (18.92%) demonstrated moderate compliance, while the majority, comprising 45 individuals (60.81%), exhibited high compliance. Furthermore, 32 patients (43.24%) identified hypoglycemia in <50% of instances, 22 patients (29.73%) recognized hypoglycemia in 50 to 70% of occurrences, and 20 patients (27.03%) acknowledged hypoglycemia in 70 to 100% of instances. Recognized hypoglycemia in the cohort was 50.00% (31.25-73.75%) of all hypoglycemia cases registered in the sensor. Data are summarized in Table SIII. CV was significantly associated with hypoglycemia events registered in the sensor (P<0.01) (Table SIV). Patients with overnight hypoglycemia had a higher CV level (P<0.01) (Table SIV).

There were no differences in compliance levels and recognition of hypoglycemia between the DKD study groups. Patients with progressive DKD marked fewer hypoglycemia events in their diary compared with those with stable DKD, but this difference was not statistically significant (P=0.08). A comprehensive summary of these findings is presented in Table III, which provides a detailed breakdown of the results.

### Correlations and regression analysis

*CGM data, clinical markers and DKD progression.* Patterns of correlations between CGM metrics and clinical variables were similar for several parameters and differed in other cases between the groups.

Correlations in the whole study group are presented in Table SIV. With regard to the kidney markers, eGFR was weakly negatively correlated with the median duration of low glucose events (ρ=-0.253; P<0.03) in the whole study group (Table SIV). However, eGFR slope and albuminuria were not statistically significantly correlated with CGM metrics in any of the study groups and neither in the whole cohort (Table SIV, Table SV and Table SVI).

**Figure 1:**
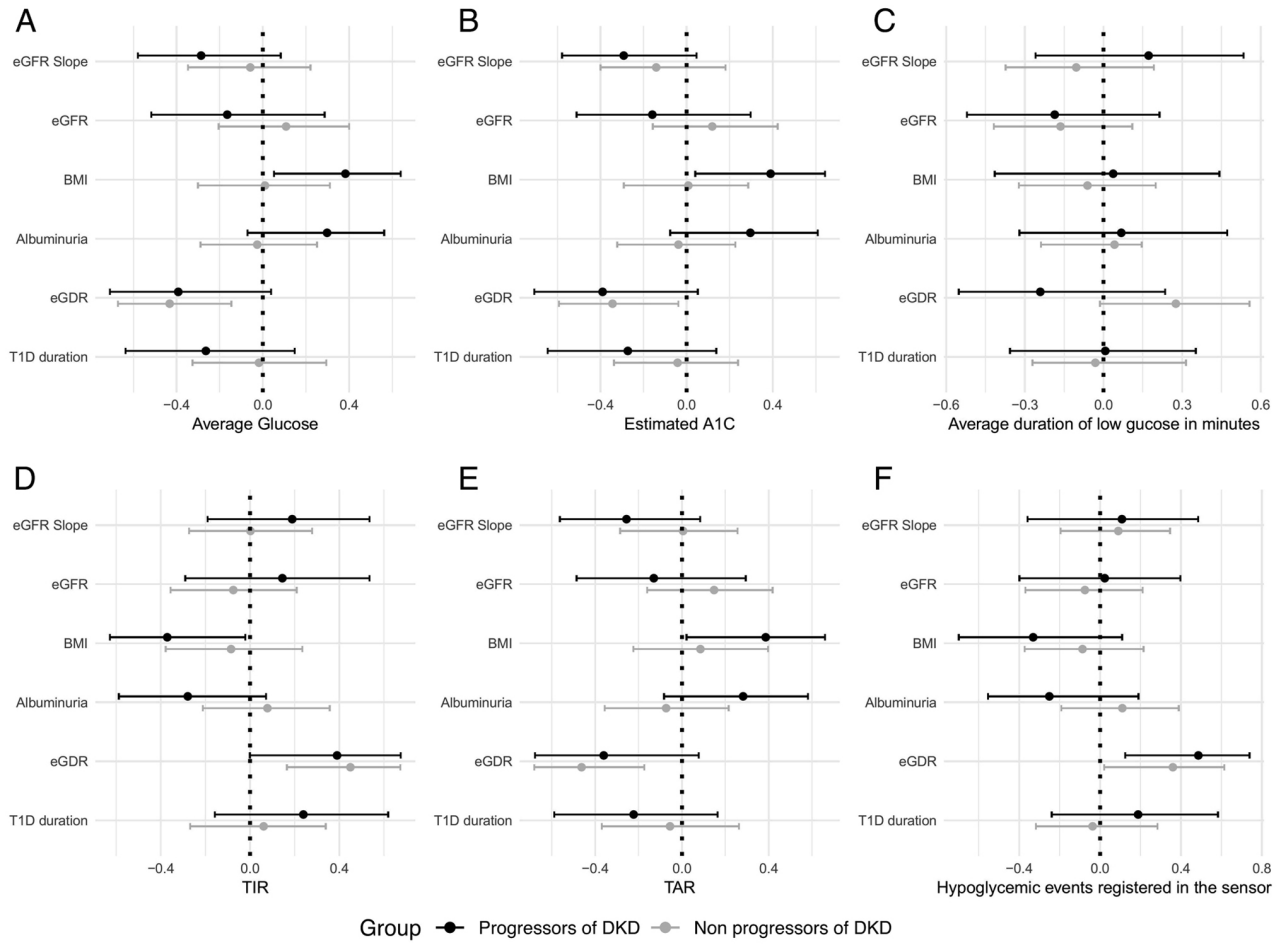
Forest plots demonstrating the correlations between continuous glucose monitoring parameters, kidney function markers, BMI, T1D duration, eGDR in both study groups. Spearman's correlation coefficients (95% confidence level) are illustrated by forest plots for (A) average glucose, (B) estimated A1C, (C) average duration of low glucose in minutes, (D) TIR, (E) TAR, and (F) hypoglycemic events registered in the sensor. The eGFR slope was calculated as a regression line using at least three eGFR calculations over the follow-up period (at least three years). BMI, body mass index; T1D, type 1 diabetes; eGDR, estimated glucose disposal rate; TIR, time in range; TAR, time above range; eGFR, estimated glomerular filtration rate.

The differences in the correlation patterns between CGM metrics and clinical factors between both study groups is highlighted in Fig. 1.

Furthermore, in the group with progressive DKD, the insulin resistance marker eGDR showed a moderate positive correlation with the number of low glucose events (ρ=0.422; P=0.03) and TIR (ρ=0.390; P=0.04), and statistically significant negative correlations with average glucose (ρ=-0.392; P=0.04), GMI (ρ=-0.406; P=0.03), and estimated A1c (ρ=-0.390; P=0.04) (Table SVI). Similar correlations were observed in the group with stable DKD (Table SV). As regards the BMI, it was negatively correlated with the number of low glucose events (ρ=-0.507; P<0.01), positively correlated with TAR (ρ=0.386; P=0.04), average glucose (ρ=0.383; P=0.04), GMI (ρ=0.406; P=0.03) and estimated A1c (ρ=0.390; P=0.04) in subjects with progressive DKD (Table SVI), but in the group with stable DKD, statistically significant correlations between these markers were not observed (Table SV). This might indicate that being overweight impacts glucose control at a greater degree in the case of DKD.

Several differences were identified in the correlation patterns of CGM metrics in the study groups (Table SIV, Table SV and Table SVI). For example, CV was significantly negatively correlated with average glucose (ρ=-0.433; P=0.02) in the progressive DKD group. In addition, TIR was significantly positively correlated with CV in the progressive DKD group (ρ=0.403; P=0.03), but not in the stable DKD group (ρ=-0.138; P=0.36) (Table SV and Table VI). Conversely, TAR and CV exhibited a negative statistically significant correlation in the progressive DKD group (ρ=-0.548; ρ<0.01; Table SVI), but not in the stable DKD group (ρ=-0.002; P=0.99; Table SV). These data indicated that the achievement of improved diabetes control is linked to an increase in GV in subjects with progressive DKD (Table SIV, Table SV, Table SVI and Table SVII).

The present study explored cross-sectional associations between glycemic indicators and the progression of DKD (Table IV). Notably, a longer TIR was associated with a significant 3% decrease in the odds of DKD progression (P=0.04). Conversely, a higher TAR had a significant 2.5% increase in the odds of DKD progression (P=0.04). The average duration of low glucose events were associated with a 0.8% increase in the odds of progression for each additional minute (P=0.05), indicating a statistically significant but borderline effect. Elevated estimated A1C levels were significantly associated with a 39.5% increase in the odds of DKD progression (P=0.03). An increase in the frequency of low glucose events was not significantly associated with DKD progression (P=0.18). These findings highlight the nuanced relationships between glycemic indicators and the likelihood of DKD progression.

## Discussion

In the present study, it was demonstrated that subjects with T1D and progressive DKD have less controlled glycemia as assessed by CGM in comparison to subjects with stable DKD. Moreover, varying correlation patterns were identified between CGM metrics and clinical markers in the study groups. Ultimately, it was determined that CGM metrics were statistically significant predictors of progressive DKD in the present study.

CGM metrics in the whole study cohort were quite poor as compared with what the guidelines recommend (10), including a median CV of 39%, a TAR of 43%, a median TIR of 51%, and overnight hypoglycemia prevalent in 74,32% of the cohort. Notable differences in glucose management were observed between patients with stable DKD and those with progressive DKD. Particularly, patients with progressive DKD demonstrated higher average glucose levels, and spent less TIR and more TAR, as compared with subjects with stable DKD. An extended duration of low glucose events were noted in patients with progressive DKD, indicating an increased risk of hypoglycemia-related complications. This finding underscores the challenges of glycemic control as DKD progresses. Several factors may be involved in this process. The patients in the progressive DKD group had longer diabetes duration, a higher prevalence of CVD and retinopathy, indicating more severe disease burden (28). As previously demonstrated by the authors of the present study, the prevalence of diabetic gastroenteropathy was higher in patients with progressive DKD (29). This complication may lead to changes in food passage through the gastrointestinal tract and abnormal absorption of nutrients. Although not assessed in this study, autonomic nervous system dysfunction which develops in numerous patients with long-standing diabetes, may cause decreased hypoglycemia awareness (30). Altogether, the aforementioned factors may complicate insulin dosing and lead to increased GV (17). On the other hand, patients with DKD are more prone to hypoglycemia due to decreased gluconeogenesis in the kidney, but intermittent hypoglycemia induces a hormonal surge, leading to renal stress and exacerbating DKD (31-33). Additionally, GV can contribute to endothelial dysfunction and increased oxidative stress, key factors in DKD pathogenesis (34,35). However, the present findings indicate that achieving adequate glucose control is challenging in subjects with progressive DKD. Indeed, it was shown that TIR was significantly positively correlated with CV, while average glucose, estimated A1C and GMI were negatively correlated with CV in patients with DKD. At the same time, such a situation is not observed in participants with stable DKD. That poses a therapeutic challenge for glucose management in patients with DKD and underlines the necessity for setting personalized glycemic targets for subjects with DKD (10).

Due to the recent introduction of CGM into diabetes care, only a few studies report data on correlations between CGM metrics and diabetes complications, which underlines the novelty of the present study. For example, associations were reported between lower TIR and higher prevalence of composite MVC (presence of neuropathy, retinopathy, or nephropathy) in 515 subjects with T1D (16). In the aforementioned study and in contrast to the findings of the present study, patients with and without diabetic nephropathy did not significantly differ in TIR. It is plausible that this finding resulted from the small number of patients with nephropathy in the study (37 out of 550). In a meta-analysis which reviewed studies devoted to an association between CGM and diabetes complications (36), six studies addressed nephropathy and only one study (37) included data on MVC in T1D related to DKD. In this study (37) microalbuminuria was assessed in conjunction with fundoscopy. Notably, only 16 out of 32 patients were classified as having MVC, and these patients had significantly higher GV calculated from CGM data compared with patients without MVC. In contrast to the findings reviewed in previous studies (36,37), the analysis in the present study focused on CGM metric comparison between DKD groups (and not MVC as a composite endpoint). The present study defined DKD progression as a combination of decreased eGFR and increased albuminuria, and it revealed the association between CGM metrics and progressing DKD, using data from the longitudinal LatDiane study. It is stressed that comparison of CGM results with regard to DKD, between studies with patients with DKD in type 2 diabetes and T1D is not possible. In addition to being two different diseases with varying treatments, courses of disease and rates of complications, the histological appearance and pathogenesis of DKD in these two diabetes types differ (38-40). In addition, there are numerous studies demonstrating associations between HbA1c variability and DKD progression, but these data cannot be compared with CGM results. A systematic review consolidating data from 14 studies with over 62,000 participants, demonstrated the link between high GV (assessed by HbA1c variability) and increased incidence and progression of chronic kidney disease, including a heightened risk of advancing to ESRD (41,42).

Considering clinical factors that were identified as being involved in glucose control in progressive DKD, higher BMI was positively correlated with longer TAR, higher average glucose, GMI and estimated A1C in subjects with progressive DKD in contrast to participants with stable kidney markers. This may indicate that being overweight impacts glucose control to a greater extent in the case of DKD. Previous studies have reported associations between metabolic syndrome, metabolic-associated fatty liver disease and visceral obesity with DKD (43,44). The present study also demonstrated a direct correlation between insulin resistance parameter eGDR and TIR in both study groups, indicating that high insulin sensitivity is linked to improved glycemic control, in agreement with previous findings (45).

In addition, longer duration of hypoglycemic events in DKD, identified in the present study as well as in other studues (18,46), might contribute to weight gain, fear of hypoglycemia and deterioration of glycemic control. On the other hand, longer hypoglycemia poses additional stress on kidneys through a hormonal surge, forming a vicious cycle between DKD progression and deterioration of glycemic control (47,48).

Dialysis represents an additional risk factor for GV in DKD due to abrupt changes in glucose and insulin concentrations during and after the dialysis session (13). These factors may have contributed to the poorer CGM metrics observed in the progressive DKD group. Among the 28 participants, 12 had ESRD, with 7 having undergone kidney transplantation and 5 receiving dialysis. Immunosuppressive treatment may cause an increase in insulin resistance resulting in poor glycemic control and CGM metrics (49).

Compliance to diabetes self-management can also impact CGM results. Compliance might be compromised with advancing diabetes due to fatigue caused by disease burden (28), impact of depression, anxiety, mild cognitive impairment, dementia (50). However, differences in compliance to diabetes self-management were not observed in the study groups during the CGM period, except for a trend to fewer marked hypoglycemia events in the progressive DKD group. Therefore, even sufficient diabetes self-management is not enough to achieve optimal glycemic control in patients with advancing DKD, and these patients have reduced hypoglycemia awareness. These patients may need more frequent structured diabetes education and sessions with a diabetes nurse and nutritionist (51), and are definite candidates for CGM (10,52). Unfortunately, numerous countries, including Latvia, still do not provide an opportunity to use CGM for all patients with T1D. The present study confirmed that patients with progressive DKD should be prioritized for this diabetes management method, along with children, pregnant individuals, and subjects with frequent hypoglycemia (53). Indeed, it was demonstrated that patients with ESRD resulting from DKD may improve glycemic control due to CGM usage (49). Although certain studies (54,55) indicate that the effectiveness of CGM to restore hypoglycemia awareness in T1D varies due to individual patient factors, CGM remains one of the most effective methods in reducing hypoglycemia-related complications in T1D.

The cross-sectional nature of the present study limits the ability to draw definitive causal conclusions as the temporal sequence of events (for example, what came first-poor daily glucose profiles or rapid progression of DKD), cannot be established. The relatively small sample size, especially in the progressive DKD group, might have influenced the results. Future research with a larger and more diverse sample would further strengthen the external validity of the present findings. In addition, CGM may have accuracy limitations, such as the delay in registering blood glucose changes in dynamic situations, however, this aspect is not that crucial in the case of diagnostic CGM which was used. By contrast, a rather short period of CGM (14 days) in the present study might not be sufficient for a thorough assessment of a daily glucose profile of a subject, constituting another limitation. The strengths of the present study include examination of CGM metrics in patients with different DKD progression statuses (adding to markedly limited studies in the field), considering diabetes self-management in the analysis of results, and identification of differences in correlation patterns between CGM metrics in subjects with stable and progressive DKD, which warrant a personalized approach to CGM interpretation and establishment of therapeutic targets in subjects with DKD.

To conclude, in the present study, the associations between CGM metrics and DKD status in T1D were revealed. The findings indicate the necessity for regular CGM in patients with progressive DKD for improvement of their glycemic control and DKD outcomes, but also call for the development of a personalized approach to CGM data interpretation and establishing therapeutic targets in these subjects.

## Supplementary Material

Study recruitment flow diagram. T1D, type 1 diabetes; eGFR, estimated glomerular filtration rate; CGM, continuous glucose monitoring; DKD, diabetic kidney disease.

Socio-demographic and disease-related data of the whole study cohort.

CGM results of the whole study cohort.

Compliance level of the whole study cohort.

Correlations in the whole study group.

Correlations in the group with stable DKD.

Correlations in the group with progressive DKD.

## Figures and Tables

**Table I:** Socio-demographic and disease-related data of the two study groups.

Characteristics	Stable DKD (N=47)	Progressive DKD (N=28)	P-value
Male, n (%)	22 (46.81%)	9 (32.14%)	0.21
Age, years	39.00 (31.50-51.00)	48.00 (36.75-54.50)	0.05
Smoking, n (%)	12 (25.53%)	8 (28.57%)	0.39
Body mass index, kg/m^2^	24.40 (22.95-27.90)	25.40 (22.42-28.62)	0.84
Duration of diabetes, years,	23.00 (13.00-27.00)	31.00 (19.25-37.00)	0.01
Hypertension, n (%)	23 (48.93%)	21 (75.00%)	0.02
Retinopathy, n (%)	19 (40.43%)	20 (71.43%)	<0.01
Severe retinopathy, n (%)	16 (34.04%)	18 (64.3%)	0.01
Autoimmune thyroid disease, n (%)	12 (25.4%)	10 (35.7%)	0.16
Other autoimmune diseases, n (%)	6 (12.76%)	8 (28.6%)	0.10
CVD, n (%)	3 (6.38%)	11 (39.3%)	<0.01
Kidney transplantation, n (%)	0 (0.0%)	7 (25.0%)	<0.01
Dialysis, n (%)	0 (0.0%)	5 (17.9%)	<0.01
HbA1c			
%	8.00 (7.20-9.45)	8.65 (7.62-9.75)	0.14
mmol/mol	64.00 (55.00-79.50)	71.0 (60.75-81.78)	
eGFR ml/min/1.73 m^2^	101.54 (85.56-114.05)	80.43 (40.78-100.58)	0.02
Albuminuria, mg/mmol	0.90 (0.45-1.85)	6.48 (2.90-70.35)	<0.01
High-density lipoprotein cholesterol, mmol/l	1.81 (1.49-2.14)	1.74 (1.42-2.17)	0.65
Low-density lipoprotein cholesterol, mmol/l	2.70 (2.17-3.40)	2.46 (1.79-3.39)	0.40
Total cholesterol, mmol/l	4.90 (4.17-5.58)	4.98 (3.98-5.98)	0.70
Triglycerides, mmol/l	0.93 (0.65-1.37)	1.32 (0.79-2.25)	0.04
Gamma-glutamyl transferase U/l	15.00 (12.00-21.50)	20.00 (14.00-30.50)	0.09
Alanine transaminase U/I	23.00 (18.00-32.50)	23.00 (16.50-30.00)	0.68
Aspartate aminotransferase, U/I	25.00 (19.00-31.00)	26.50(19.25-35.50)	0.36
C-reactive protein, mg/l	0.89 (0.49-2.66)	1.43(0.57-3.38)	0.25
Estimated glucose disposal rate, mg/kg/min	7.20 (4.50-9.25)	4.90(3.75-8.33)	0.08

Continuous variables are presented as the median (IQR). Diabetic retinopathy, history of any stage of retinopathy based on medical recordings. Arterial hypertension, systolic blood pressure ≥140 mmHg (18.7 kPa) or diastolic blood pressure ≥90 mmHg (12.0 kPa), or a history of antihypertensive drug treatment. Autoimmune thyroid disease, Hashimoto's thyroiditis or Graves' disease. Other autoimmune diseases, history of autoimmune rheumatologic disease such as rheumatoid arthritis, sacroiliitis, psoriasis and asthma. CVD, defined as a history of acute myocardial infarction, coronary bypass/percutaneous transluminal coronary angioplasty stroke, amputation, peripheral vascular disease. DKD, diabetic kidney disease; CVD, cardiovascular disease; eGFR, estimated glomerular filtration rate; IQR, interquartile range.

**Table II:** CGM results of the two study groups.

CGM	Stable DKD (N=47)	Progressive DKD (N=28)	P-value
Sensor days	15.00 (15.00-15.00)	15.00 (15.00-15.00)	0.98
Average glucose, mmol/l	8.90 (7.60-10.70)	10.50 (8.82-13.10)	0.03
Coefficient of variance, %	39.30 (35.60-42.70)	39.70 (35.70-45.80)	0.52
Glucose management indicator, %	7.10 (6.60-7.90)	7.85 (7.10-8.90)	0.03
Time above range, %	37.00 (25.00-53.00)	53.00 (35.50-68.75)	0.05
Time in range, %	56.00 (37.00-70.00)	43.00 (29.75-55.50)	0.03
Time below range, %	5.00 (6.00-11.00)	3.50 (1.00-7.75)	0.38
Low glucose events, n	11.00 (6.00-17.00)	6.00 (4.00-14.00)	0.09
Average duration of low glucose events, min	95.00 (74.00-125.00)	117.50 (87.25-171.75)	0.03
Estimated A1C, %	7.20 (6.30-8.40)	8.20 (7.20-9.90)	0.02
Overnight hypoglycemia, n (%)	37 (78.7%)	18 (66.7%)	0.25

Continuous variables are presented as the median (IQR). CGM, continuous glucose monitoring; DKD, diabetic kidney disease; IQR, interquartile range.

**Table III:** Compliance level of the two study groups.

Parameters of compliance	Stable DKD (N=47)	Progressive DKD (N=27)	P-value
Low compliance level	8 (17.0%)	7 (25.9%)	0.66
Moderate compliance level	10 (21.3%)	4 (14.8%)	
High compliance level	29 (61.7%)	16 (59.3%)	
Recognition of hypoglycemia in <50% of occurrences	22 (46.8%)	10 (37.0%)	0.83
Recognition of hypoglycemia in 50-70% of occurrences	11 (23.4%)	11 (40.7%)	
Recognition of hypoglycemia in 70-100% of occurrences	14 (29.8%)	6 (22.2%)	
Marked hypoglycemia in diary	6.00 (2.00-9.00)	3.00 (1.00-6.00)	0.08

Variables are presented as n (%). Low compliance level, entries were recorded for the necessary parameters on <6 days out of the 14-day period. Moderate compliance level, entries were recorded for the necessary parameters on at least 6 to 10 days out of the 14-day period. High compliance level, entries were recorded for the necessary parameters on at least 10 days out of the 14-day period. DKD, diabetic kidney disease.

**Table IV:** Association between the progression of DKD and CGM parameters.

Variable	Model	Hosmer-Lemeshow test	OR	95% CI	P-value
Time in range (%)	1	0.64	0.970	0.944; 0.998	0.04
Time above range (%)	2	0.72	1.025	1.001; 1.050	0.04
Average duration of low glucose events (min)	3	0.59	1.008	1.000; 1.016	0.05
Estimated A1C	4	0.99	1.395	1.040; 1.872	0.03
GMI	5	0.60	1.582	0.999; 2.505	0.05
Low glucose events	6	0.72	0.954	0.890; 1.023	0.18
Average glucose	7	0.53	1.217	0.998; 1.489	0.05

Results of the binary logistic regression analysis with the presence of progressive DKD as the response variable. Data are presented as the OR with 95% CI and P-values. All models were adjusted for sex, BMI, age and diabetes duration. DKD, diabetic kidney disease; CGM, continuous glucose monitoring; OR, odds ratio; CI, confidence interval; GMI, glucose management indicator; BMI, body mass index.

## Data Availability

The data generated in the present study are not publicly available due to privacy and ethical restrictions. However, access to the data may be requested from the corresponding author, subject to appropriate ethical approvals.
