# Supplementary material for: Glucose control and variability assessed by continuous glucose monitoring in patients with type 1 diabetes and diabetic kidney disease
Source: Biomed Rep. 2024 Dec 2;22(2):23. doi: 10.3892/br.2024.1901 (PMC11668136; doi:10.3892/br.2024.1901)

Figure S1. Study recruitment flow diagram. T1D, type 1 diabetes; eGFR, estimated glomerular filtration rate; CGM, continuous glucose monitoring; DKD, diabetic kidney disease.

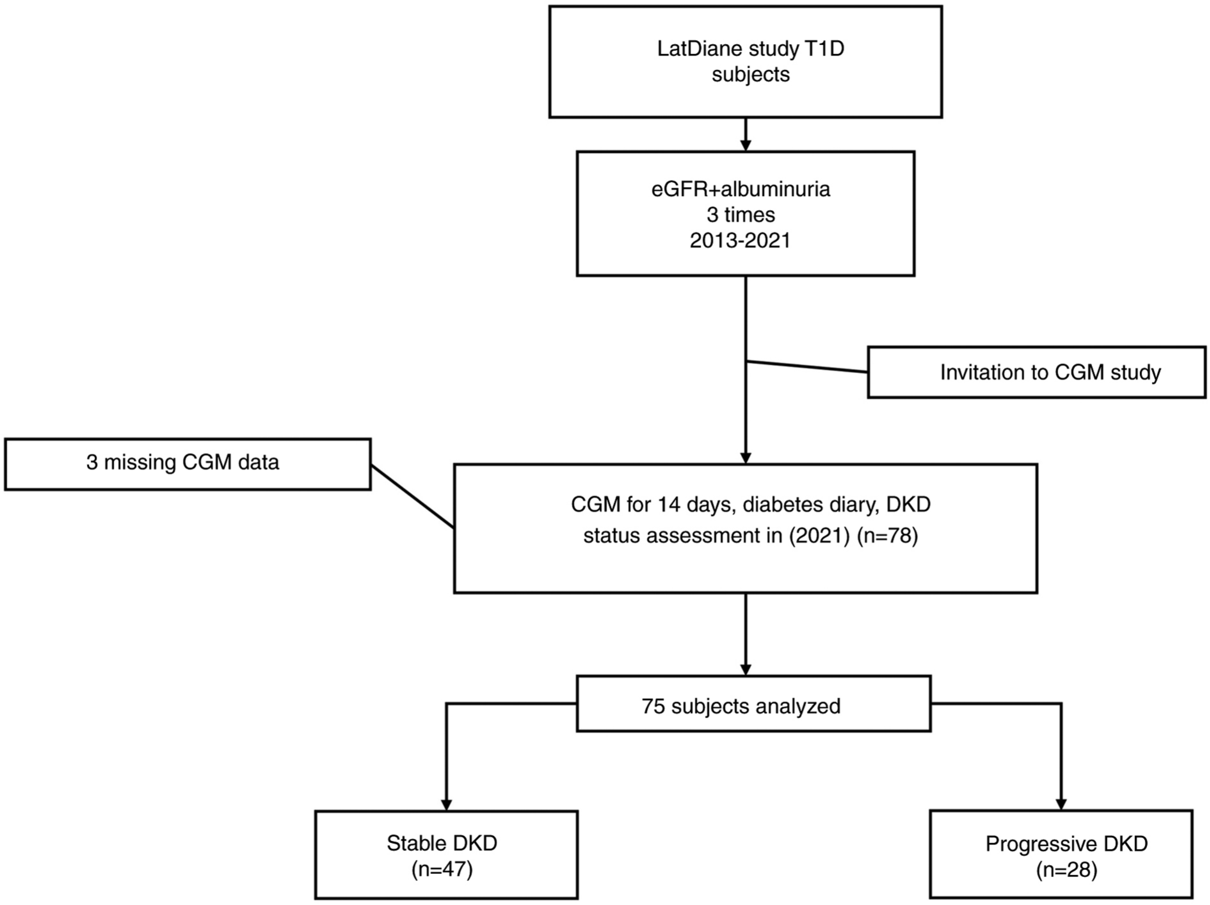

Supplement: Study recruitment flow diagram. T1D, type 1 diabetes; eGFR, estimated glomerular filtration rate; CGM, continuous glucose monitoring; DKD, diabetic kidney disease. [file Supplementary_Data1.pdf]
