# Supplementary material for: Glucose control and variability assessed by continuous glucose monitoring in patients with type 1 diabetes and diabetic kidney disease
Source: Biomed Rep. 2024 Dec 2;22(2):23. doi: 10.3892/br.2024.1901 (PMC11668136; doi:10.3892/br.2024.1901)
Supplement: Socio-demographic and disease-related data of the whole study cohort. [file Supplementary_Data2.pdf]

Table SI. Socio-demographic and disease-related data of the whole study cohort.

Characteristics	(N=75)
Cohort was follow time, years	7.46 (6.50-8.16)
Male, n (%)	31 (41.33%)
Age, years	44.00 (34.00-53.00)
Non-smokers, n (%)	41 (54.67 %)
Body mass index, kg/m^2	24.50 (22.95-28.30)
Duration of diabetes, years	25.00 (16.50-33.00)
Hypertension, n (%)	44 (58.67%)
Retinopathy, n (%)	39 (52.00%)
Autoimmune thyroid disease, n (%)	22 (29.33%)
Other autoimmune diseases, n (%)	14 (18.67%)
CVD, n (%)	14 (18.67%)
Macroalbuminuria, n (%)	10 (13.33%)
HbA1c	
%	8.20% (7.30-9.55%)
mmol/mol	66 (57-81)

Continuous variables are presented as the median (IQR).

Table SII. CGM results of the whole study cohort.

CGM	(N=75)
Sensor days	15.00 (15.00-15.00)
Average glucose, mmol/l	9.50 (8.10-11.40)
Coefficient of variance, %	39.59 (35.67-43.67)
Glucose management indicator, %	7.40 (6.80-8.20)
Time above range, %	43.00 (27.00-59.00)
Time in range, %	51.00 (37.00-66.00)
Time below range, %	4.00 (2.00-10.00)
Low glucose events, n	9.00 (4.00-15.00)
Average duration of low glucose events, min	110 (76.00-143.50)
Estimated A1C, %	7.60 (6.65-8.75)
Overnight hypoglycemia, n (%)	55 (74.32)

Continuous variables are presented as the median (IQR).

Table SIII. Compliance level of the whole study cohort.

Parameters of compliance	(N=74)
Low compliance level	15 (20.27%)
Moderate compliance level	14 (18.92%)
High compliance level	45 (60.81%)
Recognition of hypoglycemia in <50% of occurences	32 (43.24%)
Recognition of hypoglycemia in 50-70% of occurences	22 (29.73%)
Recognition of hypoglycemia in 70-100% of occurences	20 (27.03%)
Marked hypoglycemia in diary	50.00% (31.25-73.75%)

Continuous variables are presented as the median (IQR).

Table SIV. Correlations in the whole study group.

Parameter 1	Parameter 2	Correlation coefficient (ρ)	P-value
Low glucose events	BMI	-0.220	0.06
	T1D duration	0.000	->0.99
	TAR	-0.749	<0.01
	TIR	0.631	<0.01
	TBR	0.931	<0.01
	CV	0.542	<0.01
	Average glucose	-0.774	<0.01
	GMI	-0.769	<0.01
	Estimated A1C	-0.769	<0.01
	eGFR slope	0.137	0.24
	Albuminuria, mg/mmol	-0.155	0.18
	eGFR	0.063	0.59
	eGDR	0.417	<0.01
	Overnight hypoglycemia	0.390	<0.01
TAR	BMI	0.200	0.09
	T1D duration	-0.076	0.52
	TIR	-0.965	<0.01
	TBR	-0.665	<0.01
	CV	-0.184	0.12
	Average glucose	0.934	<0.01
	GMI	0.935	<0.01
	Estimated A1C	0.934	<0.01
	eGFR slope	-0.076	0.52
	Albuminuria, mg/mmol	0.176	0.13
	eGFR	-0.064	0.59
	eGDR	-0.455	<0.01
	Overnight hypoglycemia	-0.419	<0.01
TIR	BMI	-0.204	0.08
	T1D duration	0.019	0.87
	TBR	0.507	<0.01
	CV	0.044	0.71
	Average glucose	-0.885	<0.01
	GMI	-0.885	<0.01
	Estimated A1C	-0.886	<0.01
	eGFR slope	0.030	0.80
	Albuminuria, mg/mmol	-0.168	0.15
	eGFR	0.089	0.45
	eGDR	0.450	<0.01
	Overnight hypoglycemia	0.383	<0.01
TBR	BMI	-0.167	0.15
	T1D duration	0.030	0.80
	CV	0.599	<0.01
	Average glucose	-0.724	<0.01
	GMI	-0.722	<0.01
	Estimated A1C	-0.721	<0.01
	eGFR slope	0.155	0.19
	Albuminuria, mg/mmol	-0.092	0.43
	eGFR	-0.017	0.89
	eGDR	0.334	<0.01
	Overnight hypoglycemia	0.574	<0.01
CV	BMI	-0.112	0.34
	T1D duration	-0.004	0.97
	Average glucose	-0.114	0.33

	GMI	-0.113	0.33
	Estimated A1C	-0.109	0.35
	eGFR slope	0.037	0.76
	Albuminuria, mg/mmol	0.048	0.68
	eGFR	-0.050	0.67
	eGDR	0.119	0.31
	Overnight hypoglycemia	0.432	<0.01
Average glucose	BMI	0.143	0.22
	T1D duration	-0.004	0.98
	GMI	0.999	<0.01
	Estimated A1C	1.000	<0.01
	eGFR slope	-0.107	0.36
	Albuminuria, mg/mmol	0.216	0.06
	eGFR	-0.095	0.42
	eGDR	-0.453	<0.01
	Overnight hypoglycemia	-0.438	<0.01
GMI	BMI	0.145	0.21
	T1D duration	-0.012	0.92
	Estimated A1C	0.999	<0.01
	eGFR slope	-0.112	0.34
	Albuminuria, mg/mmol	0.217	0.06
	eGFR	-0.093	0.43
	eGDR	0.999	<0.01
	Overnight hypoglycemia	-0.436	<0.01
Estimated A1C	BMI	0.151	0.20
	T1D duration	-0.008	0.94
	eGFR slope	-0.107	0.36
	Albuminuria, mg/mmol	0.210	0.07
	eGFR	-0.094	0.43
	eGDR	-0.455	<0.01
	Overnight hypoglycemia	-0.438	<0.01
Average duration of low glucose in minutes	BMI	-0.025	0.83
	T1D duration	0.138	0.24
	Estimated A1C	-0.364	<0.01
	Low glucose events	0.509	<0.01
	TBR	0.702	<0.01
	TIR	0.221	0.06
	TAR	-0.364	<0.01
	GMI	-0.367	<0.01
	CV	0.501	<0.01
	Average glucose	-0.366	<0.01
	eGFR slope	0.055	0.64
	Albuminuria, mg/mmol	0.151	0.20
	eGFR	-0.253	0.03
	eGDR	0.032	0.79
	Overnight hypoglycemia	0.390	<0.01

Spearman correlation coefficient was calculated. BMI, body mass index, T1D, type 1 diabetes; TAR, time above range; TIR, time in range; TBR, time below range; CV, coefficient of variation; GMI, glucose management indicator; eGFR, estimated glomerular filtration rate; eGDR estimated glucose disposal rate.

Table SV. Correlations in the group with stable DKD.

Parameter 1	Parameter 2	Correlation coefficient (ρ)	P-value
Low glucose events	BMI	-0.083	0.58
	T1D duration	-0.040	0.79
	TAR	-0.654	<0.01
	TIR	0.518	<0.01
	TBR	0.938	<0.01
	CV	0.503	<0.01
	Average glucose	-0.689	<0.01
	GMI	-0.684	<0.01
	Estimated A1C	-0.682	<0.01
	eGFR slope	0.091	0.54
	Albuminuria, mg/mmol	0.111	0.46
	eGFR	-0.081	0.59
	eGDR	0.358	0.01
TAR	BMI	0.085	0.57
	T1D duration	-0.055	0.71
	TIR	-0.963	<0.01
	TBR	-0.587	<0.01
	CV	-0.002	0.99
	Average glucose	0.904	<0.01
	GMI	0.903	<0.01
	Estimated A1C	0.901	<0.01
	eGFR slope	0.005	0.98
	Albuminuria, mg/mmol	-0.073	0.63
	eGFR	0.148	0.32
	eGDR	-0.461	<0.01
TIR	BMI	-0.085	0.57
	T1D duration	0.061	0.68
	TBR	0.421	<0.01
	CV	-0.138	0.357
	Average glucose	-0.851	<0.01
	GMI	-0.848	<0.01
	Estimated A1C	-0.849	<0.01
	eGFR slope	0.002	0.99
	Albuminuria, mg/mmol	0.078	0.60
	eGFR	-0.075	0.62
	eGDR	0.448	<0.01
TBR	BMI	-0.015	0.92
	T1D duration	0.004	0.98
	CV	0.522	<0.01
	Average glucose	-0.686	<0.01
	GMI	-0.687	<0.01
	Estimated A1C	-0.683	<0.01
	eGFR slope	0.066	0.66
	Albuminuria, mg/mmol	0.077	0.61
	eGFR	-0.148	0.32
	eGDR	0.315	0.03
	BMI	0.020	0.89
CV	T1D duration	0.010	0.95
	Average glucose	0.071	0.64
	GMI	0.071	0.64
	Estimated A1C	0.075	0.62
	BMI	0.020	0.89
	eGFR slope	0.068	0.65

	Albuminuria, mg/mmol	0.050	0.74
	eGFR	-0.081	0.59
	eGDR	0.042	0.78
Average glucose	BMI	0.010	0.95
	T1D duration	-0.018	0.90
	GMI	0.999	<0.01
	Estimated A1C	0.999	<0.01
	eGFR slope	-0.058	0.70
	Albuminuria, mg/mmol	-0.026	0.86
	eGFR	0.108	0.47
	eGDR	-0.432	<0.01
GMI	BMI	0.002	0.99
	T1D duration	-0.021	0.89
	Estimated A1C	0.999	<0.01
	eGFR slope	-0.053	0.73
	Albuminuria, mg/mmol	-0.017	0.91
	eGFR	0.114	0.44
	eGDR	-0.429	<0.01
Estimated A1C	BMI	0.008	0.96
	T1D duration	-0.042	0.78
	eGFR slope	-0.049	0.74
	Albuminuria, mg/mmol	-0.032	0.83
	eGFR	0.108	0.47
	eGDR	-0.344	0.02
Average duration of low glucose in minutes	BMI	-0.057	0.70
	T1D duration	0.054	0.72
	Estimated A1C	-0.550	<0.01
	Low glucose events	-0.682	<0.01
	TBR	0.857	<0.01
	TIR	0.377	0.01
	TAR	-0.522	<0.01
	GMI	-0.559	<0.01
	CV	0.508	<0.01
	Average glucose	-0.552	<0.01
	eGFR slope	-0.017	0.91
	Albuminuria, mg/mmol	0.064	0.68
	eGFR	-0.210	0.16
	eGDR	0.239	0.11

Spearman correlation coefficient was calculated. DKD, diabetic kidney disease; BMI, body mass index, T1D, type 1 diabetes; TAR, time above range; TIR, time in range; TBR, time below range; CV, coefficient of variation; GMI, glucose management indicator; eGFR, estimated glomerular filtration rate; eGDR estimated glucose disposal rate.

Table SVI. Correlations in the group with progressive DKD.

Parameter 1	Parameter 2	Correlation coefficient (ρ)	P-value
Low glucose events	BMI	-0.507	<0.01
	T1D duration	0.232	0.24
	TAR	-0.854	<0.01
	TIR	0.761	<0.01
	TBR	0.904	<0.01
	CV	0.689	<0.01
	Average glucose	-0.841	<0.01
	GMI	-0.849	<0.01
	Estimated A1C	-0.844	<0.01
	eGFR slope	0.245	0.21
	Albuminuria, mg/mmol	-0.225	0.25
	eGFR	0.034	0.86
	eGDR	0.422	0.03
TAR	BMI	0.386	0.04
	T1D duration	-0.223	0.25
	TIR	-0.968	<0.01
	TBR	-0.772	<0.01
	CV	-0.548	<0.01
	Average glucose	0.971	<0.01
	GMI	0.975	<0.01
	Estimated A1C	0.972	<0.01
	eGFR slope	-0.256	0.19
	Albuminuria, mg/mmol	0.282	0.15
	eGFR	-0.130	0.51
	eGDR	-0.358	0.06
TIR	BMI	-0.371	0.05
	T1D duration	0.239	0.22
	TBR	0.633	<0.01
	CV	0.403	0.03
	Average glucose	-0.937	<0.01
	GMI	-0.941	<0.01
	Estimated A1C	-0.938	<0.01
	eGFR slope	0.189	0.34
	Albuminuria, mg/mmol	-0.279	0.15
	eGFR	0.145	0.46
	eGDR	0.390	0.04
TBR	BMI	-0.366	0.06
	T1D duration	0.146	0.46
	CV	0.741	<0.01
	Average glucose	-0.753	<0.01
	GMI	-0.756	<0.01
	Estimated A1C	-0.757	<0.01
	eGFR slope	0.268	0.17
	Albuminuria, mg/mmol	-0.181	0.36
	eGFR	0.021	0.92
	eGDR	0.279	0.15
CV	BMI	-0.258	0.19
	T1D duration	-0.001	>0.99
	Average glucose	-0.433	0.02
	GMI	-0.448	0.02
	Estimated A1C	-0.436	0.02
	eGFR slope	0.004	0.99
	Albuminuria, mg/mmol	-0.069	0.73

	eGFR	-0.062	0.75
	eGDR	0.269	0.17
Average glucose	BMI	0.383	0.04
	T1D duration	-0.262	0.18
	GMI	0.998	<0.01
	Estimated A1C	0.999	<0.01
	eGFR slope	-0.286	0.14
	Albuminuria, mg/mmol	0.298	0.12
	eGFR	-0.165	0.40
	eGDR	-0.392	0.04
GMI	BMI	0.406	0.03
	T1D duration	-0.278	0.15
	Estimated A1C	0.998	<0.01
	eGFR slope	-0.303	0.12
	Albuminuria, mg/mmol	0.302	0.12
	eGFR	-0.168	0.39
	eGDR	-0.406	0.03
Estimated A1C	BMI	0.390	0.04
	T1D duration	-0.273	0.16
	eGFR slope	-0.292	0.13
	Albuminuria, mg/mmol	0.296	0.13
	eGFR	-0.159	0.42
	eGDR	-0.390	0.04
Average duration of low glucose in minutes	BMI	0.037	0.85
	T1D duration	0.007	0.97
	Estimated A1C	-0.184	0.35
	Low glucose events	0.280	0.15
	TBR	0.541	<0.01
	TIR	0.114	0.56
	TAR	-0.251	0.20
	GMI	-0.175	0.37
	CV	0.459	0.01
	Average glucose	-0.180	0.36
	eGFR slope	0.172	0.38
	Albuminuria, mg/mmol	0.068	0.73
	eGFR	-0.186	0.34
	eGDR	-0.241	0.22

Spearman correlation coefficient was calculated. DKD, diabetic kidney disease; BMI, body mass index, T1D, type 1 diabetes; TAR, time above range; TIR, time in range; TBR, time below range; CV, coefficient of variation; GMI, glucose management indicator; eGFR, estimated glomerular filtration rate; eGDR estimated glucose disposal rate.
